# Hippocampal overexpression of Down syndrome cell adhesion molecule in amyloid precursor protein transgenic mice

**DOI:** 10.1590/1414-431X20176049

**Published:** 2017-05-15

**Authors:** Y.L. Jia, Z.X. Fu, B.H. Zhang, Y.J. Jia

**Affiliations:** 1 Department of Neurology, the First Affiliated Hospital, Zhengzhou University, Zhengzhou, Henan Province, China; 2 Department of Neurology, The Central Hospital of Kaifeng, Kaifeng, Henan Province, China

**Keywords:** Down syndrome cell adhesion molecule, Amyloid precursor protein, Transgenic mouse, Hippocampus, Dementia

## Abstract

Down syndrome cell adhesion molecule (DSCAM) is located within the Down syndrome critical region of chromosome 21. DSCAM is a broadly expressed neurodevelopmental protein involved in synaptogenesis, neurite outgrowth, and axon guidance. We previously demonstrated DSCAM overexpression in the cortex of amyloid precursor protein (APP) transgenic mice, suggesting possible regulatory interactions between APP and DSCAM. APP mice exhibit deficits in hippocampus-dependent learning and memory. In this preliminary study, we examined age-related changes in DSCAM expression within the hippocampus in 16 APP transgenic mice (1, 3, 6 and 12 months old). Hippocampus-dependent spatial memory was assessed in APP mice and age-matched wild type littermates (WTs) using the Morris water maze (MWM). The cellular distribution of hippocampal DSCAM and total expression at both mRNA and protein levels were measured by immunohistochemistry, qRT-PCR, and western blotting, respectively. APP mice exhibited spatial memory deficits in the MWM. Intense DSCAM immunoreactivity was observed in the dentate gyrus granule cell layer and hippocampal stratum pyramidale. Total hippocampal DSCAM mRNA and protein expression levels were substantially higher in APP mice than WTs at 1 and 3 months of age. Expression decreased with age in both groups but remained higher in APP mice. DSCAM is overexpressed in the hippocampus over the first 12 months of life in APP mice, but especially during maturation to adulthood. In conclusion, these results suggest an association between DSCAM and APP mice, which is characterized by neuropathology and behavioral deficits. These results provide some clues for future studies on the role of DSCAM overexpression in the precocious cognitive decline observed in APP transgenic mice.

## Introduction

Down syndrome (DS) phenotypes are determined by trisomy of a particular region of chromosome 21 known as the Down syndrome critical region (DSCR). Increased copy number of the genes in the DSCR are believed to cause the clinical manifestations of DS through overexpression of their protein products (1). Neuropathological research has identified congenital and developmental abnormalities associated with precocious dementia in DS. The former include a decrease in granular cell number in the cerebral cortex, most likely the aspinous stellate cell type (2). The DS brain exhibits decreased synaptic spine number and abnormal spine morphology from infancy through adulthood (3,4). Subjects with DS also develop senile plaques and neurofibrillary tangles in the prefrontal and hippocampal cortex with concomitant age-related dementia resembling that observed in Alzheimer's disease (AD).

Down syndrome cell adhesion molecule (DSCAM) is located within the DSCR on chromosome 21q22.2-q22.3. Sequence homology to members of the immunoglobulin superfamily (5) suggests that DSCAM functions as an adhesion molecule. DSCAM is expressed in most cells of the developing nervous system and functions in neuronal generation, migration, differentiation, growth, development (4,6–8), connectivity, axon guidance (6–8), targeting (9), dendrite recognition, self-recognition (10), fasciculation, synaptic plasticity (11), and in the proper formation of neuronal connections and neuronal networks (12). This widespread expression suggests that DSCAM overexpression disrupts brain development and synaptic plasticity (6), thereby contributing to mental retardation, precocious dementia, and peripheral nerve defects observed in DS.

In a previous study, we found that DSCAM was also overexpressed in the cortex of the amyloid precursor protein (APP) transgenic mouse model of AD (2). The finding that APP overexpression is associated with DSCAM overexpression suggests regulatory interactions between *APP* and *DSCAM*, or possibly reactive DSCAM overexpression due to the neuropathological sequelae of APP overexpression, such as deposition of AD-like plaques and neurofibrillary tangles. APP mice also exhibit age-related deficits in hippocampal learning and memory, even prior to substantial Aβ deposition (13). Thus, we examined age-related changes in DSCAM expression in the hippocampus of the APP mouse.

## Material and Methods

### Animals

APP transgenic mice (C57BL/6J-APP mice; n=16/group, 1–12 months) were obtained from the Institute of Laboratory Animal Sciences, Chinese Academy of Medical Sciences and Peking Union Medical College (CAMS&PUMC). The mice were generated using a full-length mutant human APP cDNA (695 V717I). The exogenous Aβ mainly deposits in neurons of cerebral cortex and hippocampus. The overexpression of APP695 and secretion of Aβ results in age-dependent plaque and neurofibrillary tangle formation, as well as other AD-like molecular, biochemical neuropathological, and behavior changes. Wild-type littermates (n=16) were used as controls. Mice had free access to food and water. They were kept at 23°C with a 12-h light/dark cycle. There was no death during the generation of the APP model.

The ethical standards of animal experiments were followed in accordance with the guidelines provided by World Medical Association Declaration of Helsinki on Ethical Principles for studies involving experimental animals. The study was approved by the Ethics Committee of the First Affiliated Hospital, Zhengzhou University.

### Morris water maze task

The Morris water maze was used to test hippocampal-dependent spatial reference learning and memory in the APP and WT mice. The experimental apparatus consisted of a circular water tank (diameter=140 cm; height=45 cm), containing water at 23°C to a depth of 35.5 cm and rendered opaque by adding milk powder. A platform (diameter=15 cm; height=35 cm) was submerged 1 cm below the water surface at the midpoint of one quadrant. The pool was located in a test room containing various prominent visual cues on the walls. Three training trials per day were conducted for five consecutive days. Mice were placed in the pool at one of the three quadrant starting positions (except the quadrant containing the platform). The start site was changed semi-randomly without within-day repetition for each mouse. The time required to escape onto the hidden platform was recorded. Mice that found the platform were allowed to remain on it for 30 s and then returned to the home cage. Mice that did not find the platform within 60 s were placed on the platform for 30 s. At 48 h after the final learning trial, spatial reference memory was tested on a single probe trial in which the escape platform was removed. Each mouse was allowed to swim freely for 60 s and latency of first occurrence, defined as the time taken to locate the target quadrant (that formerly contained the escape platform) for the first time, was measured as an index of spatial memory for platform location. Wrong time was defined as the number of times the mice crossed over the target quadrant in 60 s.

### Tissue preparation

APP and WT mice were anesthetized with 10% chloral hydrate by intraperitoneal injection, decapitated, and the whole brains were removed. Tissues were fixed in 4% paraformaldehyde for 2 days, embedded in paraffin, and then sliced into 4-μm sagittal sections for immunohistochemistry. For western blotting and reverse-transcription polymerase chain reaction (RT-PCR), tissue samples were obtained from the hippocampi of 1-, 3-, 6-, and 12-month-old APP and WT mice, flash frozen, and stored at -80°C until used.

### Immunohistochemistry

Paraffin-embedded sections were deparaffinized and incubated for 9 min at 90°C in 0.01 mol/L sodium citrate buffer solution, pH 6.0, for antigen retrieval. The sections were immersed in 3% hydrogen peroxide in deionized water for 10 min to quench endogenous peroxidase activity. Non-specific binding was blocked by incubation for 30 min in normal goat serum at room temperature (RT). Sections were incubated overnight at 4°C in phosphate buffered saline (PBS) containing anti-DSCAM antibody (1:50; N-16, sc-79437; Santa Cruz Biotechnology, USA) (2), followed by a 30-min incubation in biotinylated goat anti-rabbit IgG antibody (Zymed Laboratories, USA) at RT. Sections were incubated with horseradish peroxidase (HRP)-conjugated streptavidin (Zymed) for 30 min at RT. Between staining steps, the sections were thoroughly washed in PBS for 2 min. The specimens were counterstained with hematoxylin and mounted with appropriate mounting medium.

### Protein extraction and western blotting

For protein extraction, hippocampus tissue samples were thawed and homogenized in lysis buffer containing 40 mg/mL CHAPS, 50 mM Tris-HCl, pH 7.6, 0.4808 mg/mL urea, 1 mM phenylmethylsulfonyl fluoride, 5 μg/mL aprotinin (Amresco, USA), and 5 μg/mL leupeptin (Amresco). Lysates were centrifuged at 14,000 *g* for 5 min at 4°C, and the supernatants were collected. Supernatant proteins (30 or 60 μg/lane) were separated on 10% SDS-polyacrylamide gels and then electrophoretically transferred to nitrocellulose membranes (Invitrogen, USA). Membranes were blocked in 8% skim milk for 1 h at RT, then incubated in anti-DSCAM (1:1,000; N-16, sc-79437; Santa Cruz) overnight at 4°C and anti-β-actin as an internal reference (1:1,000; sc-81178; Santa Cruz). Immunolabeled membranes were then incubated in HRP-labeled goat anti-rabbit IgG (1:4,000; Zymed) for 45 min at RT. Bands were visualized using enhanced chemiluminescence and autoradiography (Hyperfilm-ECL; Amersham, Sweden). We analyzed band optical density using an image analysis system (Gel-Pro Analyzer Version 3.0, Media Cybernetics Inc., USA) and calculated the ratio of DSCAM to β-actin optical density for estimation of DSCAM expression.

### Quantitative reverse transcription-polymerase chain reaction (qRT-PCR)

Total RNA was extracted from tissue blocks using the RecoverAll Total Nucleic Acid Isolation kit for FFPE (Thermo Fisher Scientific, USA) according to the manufacturer's instructions. For determination of RNA concentration and purity, the solution (2 µL) was tested with a NanoDrop 2000 (Thermo Fisher Scientific) at absorbance 260/280. A value of 1.8-2.0 was considered acceptable. The RNA samples were stored at –80°C.

RNA was reversely transcribed using the RevertAid First Strand cDNA synthesis kit (#K1622; Thermo Fisher Scientific), according to the manufacturer's instructions, using 2 µL of RNA sample, 1 µL of oligo(dT)18 primer, 4 µL of 5× reaction buffer, 1 µL of Riboblock RNase inhibitor, 2 µL of 10 mM dNTP mix, 1 µL of RevertAid M-MuLV reverse transcriptase, and 9 µL of nuclease-free water. Reaction conditions were: 1) 25°C for 5 min; 2) 42°C for 60 min, and 3) 70°C for 5 min. The cDNA concentration was measured with the NanoDrop 2000 (Thermo Fisher Scientific). The cDNA was stored at –20°C.

The primers for qRT-PCR are shown in Table 1 and were synthesized by Shanghai Shenggong Biology Engineering Co., Ltd. (China). The reaction mixture contained 10 µL of DreamTaq Green PCR Master Mix, 1 µL of each primer, 1 µL of template cDNA, and 7 µL of nuclease-free water. Amplification was performed in a LightCycler 1.5 qRT-PCR system (Roche Diagnostics, Switzerland). The reaction conditions were: 1) 95°C for 3 min; 2) 32 cycles of 95°C for 30 s, 57°C for 30 s, and 72°C for 35 s, and 3) 72°C for 10 min. β-actin was used as the internal reference. Each sample was tested six times and the average value was used for analysis. The 2^-ΔΔCt^ method was used to represent the relative expression of the target gene.

**Table 1.** Primer sequences.

Gene	Primer sequence
*DSCAM*	
Forward	5′-CCACCTTACCTCAGCGAGAG -3′
Reverse	5′- TTTGCGTAGGGATTGTTTCC-3′
*β-actin*	
Forward	5′-AGGGGAGAGCGGGTAAGAGA-3′
Reverse	5′-GGACAGGACTAGGCGGAACA -3′

### Statistical analysis

SPSS 10.0 (IBM, USA) was used for all analyses. Results are reported as means±SD. One-way analysis of variance was used to compare group means followed by least significant difference (LSD) *t*-tests for pair-wise comparisons. A P<0.05 was considered to be statistically significant.

## Results

### Learning and memory functions

**Figure 1.**
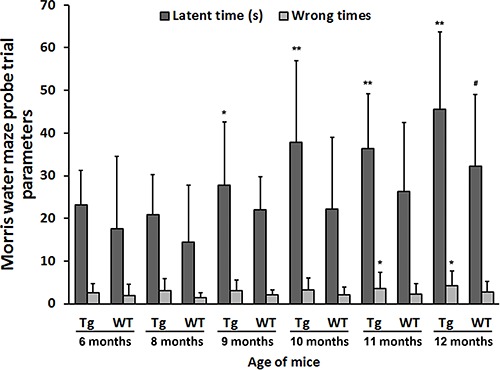
Morris water maze probe trial parameters of amyloid precursor protein transgenic mice (Tg) and age-matched wild-type littermates (WT) (n=16/group). Data are reported as means±SD. *P<0.05, **P<0.01 *vs* 6-month-old Tg mice, ^#^P<0.05 *vs* 6-month-old WT mice (ANOVA followed by least significant difference *t*-tests for pair-wise comparisons).

We first confirmed that the APP transgenic mice examined in this study exhibited age-dependent deficits in hippocampus-dependent spatial reference memory, as shown previously (13). Spatial memory was assessed in the Morris water maze by a single probe trial measuring the mean time to find the quadrant formerly containing the escape platform (target quadrant). APP mice demonstrated significantly longer search times compared with age-matched WTs for all age groups tested (Figure 1).

### DSCAM expression in mice

**Figure 2.**
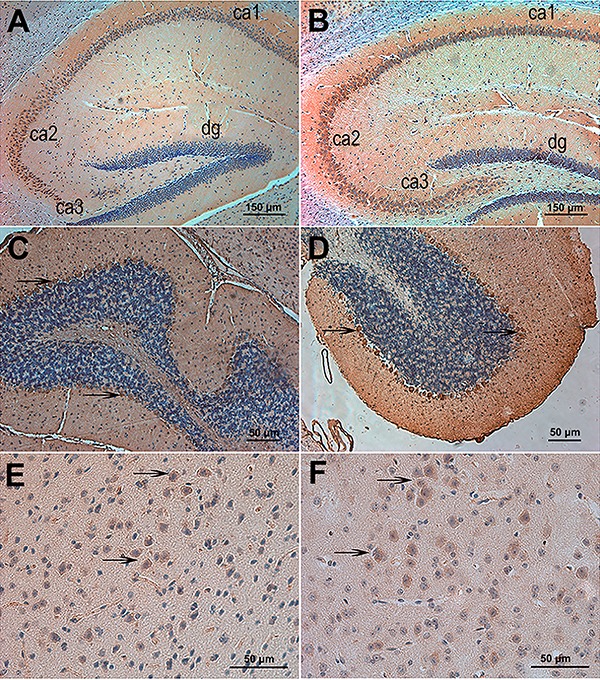
Immunohistochemistry of mouse Down syndrome cell adhesion molecule (DSCAM) in sagittal brain sections from 3-month-old mice. DSCAM is expressed in the hippocampus of (*A*) wild-type (WT) and (*B*) amyloid precursor protein (APP) transgenic mice. ca1, ca2, ca3: CA1, CA2 and CA3 regions of Ammon's horn; dg: dentate gyrus. DSCAM expression in cerebellar Purkinje cell layers of (*C*) WT and (*D*) APP transgenic mice (indicated by arrows). DSCAM expression in middle-layer pyramidal neurons (arrows) of cerebral cortex of (*E*) WT and (*F*) APP transgenic mice. Brown staining is DSCAM; blue staining is hematoxylin.

Immunohistochemistry of brain sections revealed that DSCAM is highly expressed in the cerebral cortex, granule layer of the dentate gyrus, pyramidal cell layer of Ammon's horn of the hippocampus (stratum pyramidale), thalamus, and cerebellar Purkinje cells of both WT (Figure 2A,C,E) and APP transgenic mice (Figure 2B,D,F).

### DSCAM expression in the hippocampus by age and genotype

**Figure 3.**
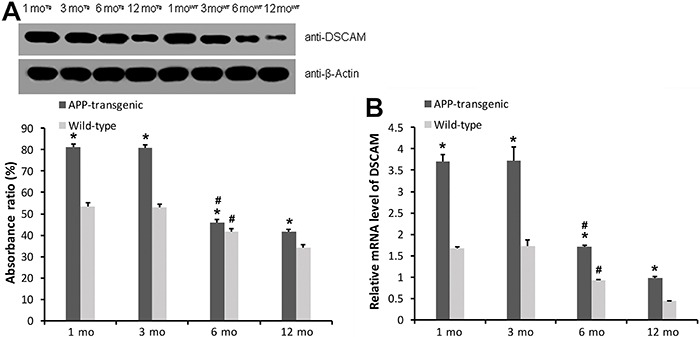
Expression of Down syndrome cell adhesion molecule (DSCAM) mRNA and protein in hippocampus of mice in different groups. DSCAM expression was measured by western blot (*A*) and qRT-PCR (*B*) in hippocampal lysates from amyloid precursor protein (APP) transgenic (Tg) and wild-type (WT) mice (1, 3, 6, and 12 months [mo] old). *P<0.05 *vs* the age-matched WT group; ^#^P<0.05 *vs* 3- and 12-mo mice of the same genome type (n=10) (ANOVA followed by least significant difference *t*-tests for pair-wise comparisons).

RT-PCR (Figure 3B) and western blotting (Figure 3A) demonstrated substantial overexpression of *DSCAM* mRNA and protein in the hippocampus of APP mice compared with WTs during maturation to adulthood (1 and 3 months of age; P<0.05). The results of qRT-PCR showed that DSCAM mRNA expression in transgenic mice was higher than that in wild-type mice of the same age (P<0.05). Intragroup comparison showed that DSCAM mRNA expression was higher at 1 and 3 months compared with that observed at 6 and 12 months (P<0.05), while the difference was not significant between 1 and 3 months (P>0.05; Figure 3).

## Discussion

In this study, it was found that DSCAM was markedly overexpressed in the hippocampus of APP transgenic mice, suggesting that dysregulation of DSCAM may contribute to the neuropathological and behavioral phenotype of APP mice. Indeed, these mice demonstrate deficits in learning and memory even before detectable plaque formation (13), during which hippocampal DSCAM expression is elevated by ∼50% relative to levels in WT mice. Moreover, DSCAM expression was enriched in highly plastic hippocampal neurons critical for learning and memory (14). In *Aplysia californica*, postsynaptic overexpression of a truncated DSCAM containing the intracellular domain blocked the clustering of α-amino-3-hydroxy-5-methyl-4-isoxazolepropionic acid (AMPA) receptors, which are necessary for expression of long-lasting forms of synaptic plasticity that underlie learning (15). Thus, overexpression of DSCAM may contribute to certain APP phenotypes, although additional studies in mice selectively overexpressing DSCAM are needed to test this hypothesis.

DSCAM immunoreactivity has been found in the core of senile plaques and in surrounding synapses, suggesting some role for DSCAM in plaque formation (16). In addition, DSCAM overexpression may contribute to DS pathogenesis through inhibitory action on synaptogenesis and neurite outgrowth (4,8–12,17–22). In fact, DSCAM trisomy was found in a human DS patient with no signs of AD-like neuropathology (23), suggesting that DSCAM overexpression alone may lead to cognitive deficits, but additional studies are necessary since this patient was also trisomic for other genes on chromosome 21.

In the present study, DSCAM was highly expressed in the cerebral cortex, hippocampus, thalamus, cerebellar Purkinje cells, and brainstem neurons of APP transgenic mice (2), which are regions involved in a variety of higher-order functions such as somatosensory information processing, voluntary movement, motor learning, explicit learning, and memory. Hippocampal DSCAM expression was significantly higher in APP transgenic mice compared with age-matched WT controls, particularly during young adulthood. Explicit memory depends on intact fibers linking dentate granule cells with hippocampal pyramidal cells as well as pathways linking the hippocampal formation with other regions such as the frontal cerebral cortex. Both over- and under-expression of the *Drosophila* DSCAM homolog resulted in defective axon guidance and abnormal neural network formation (24). That neurons may be particularly sensitive to *DSCAM* dosage is further suggested by the finding that 10-13% of embryos heterozygous for *DSCAM* show defects in Bolwig's nerve axon guidance (24). In addition, *A. californica* and *Drosophila melanogaster* indicate that the increased production of neural proteins of the immunoglobulin superfamily inhibits synapse growth or migration under some conditions (25). It is thus possible that elevated DSCAM may contribute to the abnormal gross architecture and cortical lamination defects seen in DS patients (26).

We propose that hippocampal DSCAM overexpression may be involved in learning and memory defects in APP transgenic mice, possibly by altering the adhesive properties of neural cells, inhibition of synaptogenesis and/or neurite outgrowth, disturbed axon guidance, and the proper formation of neuronal connections. In addition, DSCAM may facilitate Aβ plaque formation or enhance the neurodegenerative potential of these inclusions.

This study is not without limitations. Even if an association were observed between DSCAM expression and APP mice, this study was not designed to assess the cause-effect relationship leading to this association. Studies are warranted to investigate whether DSCAM is overexpressed in the hippocampus of DS and AD patients, how APP and DSCAM interact at both the expression and functional levels, and if and how DSCAM alone alters hippocampal synaptoplastic processes underlying learning and memory.

These results suggest an association between DSCAM and APP mice, which is characterized by neuropathology and behavioral deficits. These results provide some clues for future studies on the role of DSCAM overexpression in the precocious cognitive decline observed in APP transgenic mice.
